# Soy Infant Formula may be Associated with Autistic Behaviors

**DOI:** 10.4172/2165-7890.1000120

**Published:** 2013-11-18

**Authors:** Cara J. Westmark

**Affiliations:** Department of Neurology, University of Wisconsin, USA

**Keywords:** Autism, Autistic behavior, Phytoestrogen, Soy

## Abstract

The effects of soy-based infant formulas on childhood development are not well understood. This exploratory study evaluates the severity of autistic behaviors in association with the use of soy-based infant formula in a population of high-functioning autistic children. Medical record data were analyzed from the Simons Foundation Autism Research Initiative Simplex Collection, which included data on infant formula use and autism diagnostic scores for 1,949 autistic children. We found exploratory associations between the use of soy-based infant formula and several autistic behaviors as assessed by line-item analysis of the Aberrant Behavior Checklist, Autism Diagnostic Interview-Revised and Autism Diagnostic Observation Schedule. This study provides preliminary data that the use of soy-based infant formula may be associated with specific autistic behaviors.

## Introduction

Autism is a cluster of complex neurobiological disorders known as autism spectrum disorders (ASDs) that normally present in the second or third year of life. The core features include impairments in social interaction and communication, and repetitive stereotyped behavior [1]. Many autistic children are mentally retarded and half exhibit marked delay in motor milestones. ASDs are estimated to occur in 1 in 88 children with prevalence 4.7-fold higher in males [2]. Genetic as well as environmental factors likely contribute to the etiology of ASDs [3–5]. Recent findings in a fragile X syndrome (FXS) rodent model indicate that soy ingestion during postnatal development significantly increases seizure propensity in *Fmr1^KO^* mice [6]. FXS is the leading known genetic cause of autism accounting for approximately 5% of cases [7] with 67% of males and 23% of females with FXS meeting the diagnostic criteria for autism [7,8]. Seizure disorder, or epilepsy, is the most common co-morbidity in ASD occurring in 11–39% of cases [9]. Thus, we asked if soy-based infant formula was associated with ASD prevalence or severity. Although nearly a quarter of infant formulas are soy-based [10], much remains to be learned regarding their effects on childhood development [11–17]. Soybeans are rich in numerous bioactive compounds including saponins, protease inhibitors, phytic acid and phytoestrogens [18]. Soy-based infant formula contains high levels of phytoestrogen approaching 4.5–8 mg/kg/day [19,20]. Considering body weight, these infants are getting six to 11 times the dose of phytoestrogens necessary to exert hormone-like effects in adults [19]. The phytoestrogen daidzein has been identified as a seizure-promoting ingredient in mice [6], and the use of soy-based infant formula is associated with increased seizure incidence in autistic children [21]. This exploratory study examines associations between the use of soy-based infant formula in autistic children and line-item behaviors on autism diagnostic exams. Data was attained from medical records from a population of high-functioning autistic children in the Simons Foundation Autism Research Initiative (SFARI) Simplex Collection.

## Materials and Methods

### Participants

SFARI in collaboration with medical centers across North America collected high quality phenotype data and biospecimens from 2,644 autism simplex families. A simplex family is one in which only one child (the proband) is on the autism spectrum, while both biological parents and all siblings are not. All collection sites used the same inclusion and exclusion criteria, administered the same instruments and followed the same protocols in collecting biospecimens. Families were recruited from a coalition of clinics located at Baylor College of Medicine, Children’s Hospital of Boston, Columbia University, Emory University, McGill University, University of California-Los Angeles, University of Illinois at Chicago, University of Michigan, University of Missouri, University of Washington, Vanderbilt University and Yale University.

The inclusion criteria included proband age and a diagnosis of an ASD. The proband in the family was between four years and 17 years and 11 months of age when the phenotype measures were administered and the data collected. On the Autism Diagnostic Interview-Revised (ADI-R), the proband was required to meet one of the following criteria: (1) standard cutoff on the social and communication domains, (2) standard cutoff on the social domain and within two points of communication cutoff, (3) standard cutoff of the communication domain and within two points of social cutoff, or (4) within one point of the standard cutoffs for both the social and communication domains. On the Autism Diagnostic Observation Schedule (ADOS), the proband must have received a valid and reliable administration and must have met the cutoffs for autism spectrum disorders or autism. On the Mullen Scales of Early Learning, the Differential Ability Scales-II, the Wechsler Intelligence Scale for Children-IV or the Wechsler Abbreviated Scale of Intelligence, the proband must have had a nonverbal deviation or ratio IQ score greater than or equal to 60 (four years of age) or greater than or equal to 40 (between five and eight years of age). Participants eight years of age or older must have had a nonverbal mental age of 36 months or older. The proband was also required to have a clinical “Best Estimate Diagnosis” of autistic disorder, Asperger’s disorder, or pervasive developmental disorder not otherwise specified made by a psychologist or physician.

The exclusion criteria included: (1) pregnancy and birth issues for probands including fewer than 36 weeks gestation and less than 2,000 grams at birth, or a history of maternal pregnancy or birth complications; (2) other disorders or limitations in the proband including a positive diagnosis for FXS or Down syndrome, sensory or motor difficulties that would preclude valid use of diagnostic instruments, or a history of severe nutritional or psychological deprivation; (3) sibling diagnosed with an autism spectrum disorder, mental retardation (except Down syndrome), schizophrenia, or a psychiatric disorder requiring treatment with more than one psychotropic medication; (4) sibling with an Adaptive Behavior Standard score on the Vineland-II that was 70 or below or an Individualized Education Plan for extensive special education services; (5) parent diagnosed with an autism spectrum disorder, mental retardation, or schizophrenia; or (6) any second- or third-degree relative diagnosed with an autism spectrum disorder.

### Procedure

Medical record data for the autistic probands and family members were available from the Simons Simplex Collection (SSC) through an interactive database that facilitated correlations between clinical, genetic, and neurobiological data [22]. The dataset utilized for this study was from SSC version 14 Public Cohort, released March 21, 2012 (http://sfari.org/resources/sfari-base). The proband study participants exhibited moderate to severe autistic symptoms with relatively little intellectual disability. Data regarding the use of soy-based infant formula were obtained from the medical history form, a questionnaire regarding the proband and administered to the parent by the clinical research staff. Specifically, parents were asked, “Type of Formula used” with the options of “Soy, Cow’s Milk Based and Other/specify”. Data regarding autism diagnostic scores were obtained from Aberrant Behavior Checklist (ABC), ADI-R and ADOS testing. In all exams, higher test scores indicate greater behavioral problems in the specified areas. Means and standard deviations were computed to describe the cohorts. Statistical significance was determined by Student t-test analyses with two-sided P values of 0.05 regarded as statistically significant. Adjustments for multiple comparisons were not universally applied since the analyses were considered exploratory in nature.

### Diagnostic tests

The ABC is a symptom checklist for assessing the severity of problem behaviors in children and adults with intellectual disability [23]. There are 58 items on the ABC, which are categorized into 5 subscales including: (I) irritability/agitation (15 questions); (II) lethargy/social withdrawal (16 questions); (III) stereotypic behavior (7 questions); (IV) hyperactivity/noncompliance (16 questions); and (V) inappropriate speech (4 questions) [24]. The assessment takes between 10–15 min to complete with each item rated on scale from 0 (not a problem) to 3 (problem is present to a severe degree). Scores to individual questions are added and presented as sub-scale scores.

The ADI-R is a structured interview conducted with parents of individuals who have been referred for the evaluation of ASD [25]. The ADI-R is based on the Diagnostic and Statistical Manual of Mental Disorders, 4^th^ Edition (DSM-IV) and the International Classification of Diseases, 10^th^ Revision (ICD-10) criteria for autism and pervasive developmental disorders, and contains 93 questions regarding children’s early development, communication, social interaction, and patterns of behavior. The ADI-R can be used for diagnosis of subjects with a mental age of at least 18 months, is usually conducted by a psychiatrist or licensed professional and generally takes 1–2 hours. Scores to individual questions are determined by the interviewer based on their evaluation of their parent’s response using a rating scale of 0 (behavior of the type specified in the coding is not present), 1 (behavior of the type specified is present in an abnormal form, but not sufficiently severe or frequent to meet the criteria for a 2), 2 (definite abnormal behavior), 3 (extreme severity of the specified behavior), 7 (definite abnormality in the general area of the coding, but not of the type specified), 8 (not applicable), and 9 (not known or asked). Total scores are calculated for each of the behavioral areas where an individual score of 3 collapses to 2 and scores of 7, 8 or 9 drop to 0. A diagnostic algorithm provides cut-offs in each of the three domains [Reciprocal Social Interaction, cutoff=10; Communication and Language, cutoff=8 (if verbal), cutoff=7 (if nonverbal); and Restricted, Repetitive and Stereotyped Patterns of Behavior, cutoff=3]. The ADI-R examines the functioning of the child in the past (most aberrant, ever) and the present. Caregivers are asked questions regarding the greatest impairment noted between the ages of 4 to 5 or ever in their child’s lifetime as well as the current degree of impairment.

The ADOS is an observational, open-ended assessment in which an examiner uses a series of situations and interview questions to assess communication, social interaction, play and imagination for the diagnostic evaluation of individuals suspected of having ASD [26]. Dependent on subject age and verbal skill, one of four ADOS modules is utilized. Module 1 is used with children who do not consistently use phrase speech, Module 2 with those who use phrase speech but are not verbally fluent, Module 3 with verbally fluent children, and Module 4 with verbally fluent adolescents and adults. The ADOS cannot be used with nonverbal adolescents and adults. Regardless of the module administered, criteria are scored on a 4-point scale in the areas of Communication and Social Interaction (CSI), Restricted and Repetitive Behavior (RRB) and Social Affect. The scoring scale is the same as previously described for the ADI-R. The test takes 30–45 minutes.

### Ethical standards

All human studies conducted by the SSC were approved by the Institutional Review Board at Columbia University Medical Center, and have been performed in accordance with the ethical standards laid down in the 1964 Declaration of Helsinki and its later amendments. All guardians or research subjects provided written informed consent. The privacy of participants was protected by using global unique identifiers. The research protocol for using the SSC in this study was approved by the Human Research Protection Program at the corresponding author’s university, which determined that the study qualified for exemption.

## Results

### Patient demographics

We utilized medical record data available from SFARI [22] for this retrospective analysis of autistic behaviors in response to soy-based infant formula. The study population was defined as all probands in the SSC with non-null medical record data regarding the type of infant formula used. The demographics regarding this study population have been previously described and indicate that soy-based formula was utilized in 17.5% of the study population and females comprised 13.4% of the 1,949 subjects [21]. The 6.5-fold increase in the number of male subjects is supported by recent epidemiological data indicating that autism is 4.7-fold more prevalent in boys than girls [2]. There is a 2.6-fold higher rate of febrile seizures, a 2.1-fold higher rate of epilepsy comorbidity and a 4-fold higher rate of simple partial seizures in the autistic children fed soy-based infant formula [21]. Seizures are highly comorbid with ASD [9]; thus, we asked if the use of soy-based infant formula was also associated with autism severity by comparing autism behavior scores from the ABC, ADI-R and ADOS autism diagnostic exams.

### Aberrant behavior checklist

ABC exams were completed by the parent regarding the proband. Although, Total ABC scores were not statistically different between soy and non-soy cohorts, subscale (Table 1) and line item (Table 2) scores indicate that several autistic behaviors may be affected in a soy-dependent manner. Specifically, subscale 5 scores (inappropriate speech) increased one grade from 3.4 ± 2.9(SD) in the non-soy female cohort to 4.4 ± 3.2(SD) in the soy cohort (*P* ≤ 0.05). Likewise, subscale 1 scores (irritability) increased from 11 ± 8.7(SD) to 12 ± 9.0(SD) in males, which approached statistical significance (*P* ≤ 0.07). Line item analyses indicated statistically significant differences in response to soy for question #34 in males (increased incidence of cries over minor hurts) and questions #42 and #24 in females (increased incidence of prefers to be alone and decreased incidence of uncooperative, respectively). Additional line-item behaviors that approached statistical significance (P≤0.1) in males included increased severity in question #25 (depressed mood), question #29 (demands must be met immediately), question #47 (stamps feet/bangs objects/slams doors,) question #57 (temper outbursts if not own way) and question #3 (listless/sluggish/inactive). Additional line-item behaviors that approached statistical significance (*P* ≤ 0.1) in females included increased severity in question #34 (cries over minor hurts), question #52 (physical violence to self), question #30 (isolates self), question #43 (does not try to communicate), question #45 (waves or shakes extremities repeatedly), and question #22 (repetitive speech) as well as decreased severity in question #21 (disturb others). We have not applied adjustments for multiple comparisons as the analyses were exploratory in nature and not sufficiently powered. None of the line-item differences would be considered statistically significant if corrected for multiple comparisons within the individual subscales.

### Autism diagnostic interview-revised

Similarly, Total ADI-R scores were not statistically different between the soy and non-soy cohorts, although there was a statistically significant increase in the Total Restricted, Repetitive and Stereotyped Behavior (RRSB) score in males [increase from 6.4 ± 2.5(SD) in the non-soy cohort to 6.9 ± 2.4(SD) in the soy cohort, *P* ≤ 0.01] (Table 3). The specific deficit in the RRSB score was due to sub-category C1 (encompassing pattern), which increased from 1.9 ± 1.2(SD) to 2.1 ± 1.2(SD) (P≤0.01) (Table 4). This result remains statistically significant after application of a multiple comparison correction factor accounting for the 4 subscores within the RRSB domain. In females, sub-category B3 (idiosyncratic speech) approached statistical significance increasing from 3.9 ± 1.8(SD) to 4.5 ± 1.5(SD) (*P* ≤ 0.10). Increased severity was observed in 14 of 78 ADI-R line-item behaviors in the male soy cohort including question #14 (loss of communicative intent), question #29 (comprehension of simple language-most abnormal), question #52 (showing and directing attention-current), question #56 (quality of social overture-current), question #58 (inappropriate facial expressions-current), question #67 (unusual preoccupations-current), question #70 (compulsions/rituals-current), question #71 (unusual sensory interests-current), question #72 (undue general sensitivity to noise-current), question #73 (abnormal/idiosyncratic/negative response to special sensory stimulation-current), question #74 (difficulties with minor changes routines/personal environment-current and ever), question #78 (other complex mannerisms or stereotyped body movements-current), and question #83 (self-injury-current), while question #44 (head shaking-most abnormal) was improved (Table 5). In females, question #14 (loss of communicative intent) and question #55 (offering comfort-current) were more severe and question #50 (direct gaze-most abnormal) was improved in the soy cohort. ADI-R line item behaviors that retained statistical significance after correction for multiple comparisons included greater deficits in question #70 (sub-category C2, *P*=0.024), question #71 (sub-category C4, *P*=0.016) and question #78 (sub-category C3, *P*=0.0008) in males and improvement in question #50 (sub-category A1, *P*=0.016) iO females. Overall, exploratory soy-associated deficits cluster in the areas of communication for males and females as well as hypersensitivity to sensory stimulation in males.

### Autism diagnostic observation schedule

There were no statistically significant differences in Total, Communication and Social Interaction (CSI), Restricted and Repetitive Behavior (RRB) or Social Affect scores in the ADOS (Table 6). However, 4 of 29 line-item behaviors in Module 1 were more severe in the male soy cohort including frequency of vocalization directed to others, pointing, requesting, and response to joint attention (Table 7). In addition, more severe integration of gaze/other behaviors and spontaneous initiation of joint attention approached statistical significance, whereas intonation of vocalization/verbalizations approached improvement (*P* ≤ 0.08). The female soy cohort exhibited more severe scores in giving and improvement in spontaneous initiation of joint attention, and approached statistically significance in improvement in overall level of nonechoed language in Module 1. In Module 2, the male cohort exhibited more severe quality of social overtures and imagination/creativity symptoms. The female soy cohort exhibited more severe anxiety but improvement in the amount of social overtures/maintenance of attention, pointing, descriptive conventional instrumental or informal gestures, response to name, spontaneous initiation of joint attention and overall quality of rapport (Table 8). The increase in anxiety was statistically significant after correction for multiple comparisons (sub-category E3, *P*=0.005). In Module 3, the male soy cohort exhibited improvement in offers information, asks for information, reporting of events and insight and approached statistical significance for improvement in quality of social overtures, overall quality of rapport and hand/finger and other complex mannerisms (Table 9). The female soy cohort showed more severe behavior in offers information and descriptive/conventional/instrumental/informal gestures, and approached statistical significance for improvement in referencing highly specialized topics/objects/repetitive behaviors. The improvement in offers information (sub-category A5, *P*=0.002) is statistically significant after correction for multiple comparisons. There were few subjects assessed by Module 4 and none of the sub-scores were statistically different between soy and non-soy cohorts (Table 10).

## Discussion

### Autism diagnostic scores

Behavior problems are a primary concern for the caregivers of children with ASDs. Several diagnostic tests are used to screen for autistic behaviors including the ABC, ADI-R and ADOS. This exploratory analysis of individual behaviors by these autism diagnostic tests provides the opportunity to identify specific ASD symptoms that may be associated with the use of soy-based infant formula and that deserve further investigation.

In the SFARI population under study, analyses of the ABC results indicated that irritability symptoms may be more severe in the male soy cohort with 5 of the 15 line-item scores as well as the total Sub-Scale 1 score exhibiting statistically or near-statistically significant differences (unadjusted for multiple comparisons). In the female soy cohort, inappropriate speech may be more severe with the total Sub-Scale 5 score significantly different from the non-soy cohort and all 4 line-item symptoms comprising Sub-Scale 5 approaching statistical significance (*P* ≤ 0.22). In females, several items in Sub-Scale 2 (lethargy) including isolating self, preferring to be alone and does not try to communicate may be more severe in the female soy cohort while there could be a decreased incidence of Sub-Scale 4 (hyperactivity) symptoms including disturbs others and is uncooperative. The only ABC line-item symptom more severely affected in both males and females was cries over minor hurts.

In the ADI-R, male and female soy cohorts exhibited more severe exploratory deficits related to communication while males also showed hypersensitivity to sensory stimulation. The ADI-R is a much more detailed, extensive examination of parental views regarding their child’s ASD behaviors than the ABC suggesting that deficits in communication and increased hypersensitivity to sensory stimulation reported in the ADI-R are supported by increased inappropriate speech and irritability scores on the ABC. The ADI-R examines the functioning of the child in both the past and the present. Interestingly, several behaviors in the male soy cohort appeared more significantly worse in the present but not the past analyses including showing and directing attention, inappropriate facial expressions, unusual sensory interests, undue general sensitivity to noise, and abnormal idiosyncratic negative response to special sensory stimulation. Additional symptoms appeared more severe in response to soy in both the present and past analyses including quality of social overtures, compulsions/rituals, difficulties with minor changes to routines and personal environment, and self-injury. These data suggest that consumption of soy-based infant formula may affect autistic behaviors long after formula use is discontinued.

Line-item analyses of the ADOS modules indicated variability in autistic behaviors dependent on verbal fluency and gender. In Modules 1 and 2, the male soy cohorts exhibited elevated scores related to communication skills while the female soy cohorts had decreased scores, but the reverse effect was observed with Module 3 (improved communication in males and worse skills in females). Modules 1 and 2 are used in children who are not verbally fluent and worse communication in the male soy cohort was in terms of frequency of vocalizations toward others, pointing, requesting and response to joint attention (Module 1) and quality of social overtures (Module 2), whereas the female soy cohort exhibited better spontaneous joint attention, amount of social overtures, pointing, informal gestures and overall quality of rapport compared to respective non soy cohorts. Module 3 tests verbally fluent children and showed that the female soy cohort was worse in verbal (offers information) and nonverbal (informal gestures) communication while the male soy cohort exhibited better verbal communication (offers information, asks for information, reporting of events and insight) compared to their respective non-soy cohorts. As the study population was split among the four ADOS modules dependent on age and verbal fluency of the subjects, the sample size was small for each of the female soy cohorts (N=14 for Module 1, N=11 for Module 2, N=19 for Module 3 and N=0 for Module 4); however, statistically significant differences were still attained for several variables included in Modules 1–3. Overall, the ADOS data suggest a possible gender-specific response in terms of verbal and nonverbal communication in autistic children in association with the use of soy-based infant formula. Deficits in communication in the ADOS Module 3 in the female soy cohort are in agreement with communication deficits in the ABC (inappropriate speech) and ADI-R (idiosyncratic speech and loss of communicative intent). Of note, the female soy cohort also exhibited the highest rates of febrile seizures (9%) and simple partial seizures (2%) [21]. Epileptiform activity has been associated with language regression in ASD [27], but it remains to be determined if seizures cause language regression and cognitive decline or if these phenotypes share an underlying neuropathology with autism.

### Autism and diet

There is a paucity of studies examining the role of diet on autistic behaviors. A PubMed search with the terms “aberrant behavior checklist AND diet” produced only 3 results, “ADI-R AND diet” produced 0 results, and “autism AND ADOS AND diet” produced 1 result. These published studies tested cholesterol supplementation, phenylalanine-restriction, omega-3 fatty acid supplementation and a gluten- and dairy-free diet on autistic behaviors. Specifically, a double-masked, placebo-controlled, crossover trial tested the hypothesis that dietary cholesterol supplementation would have rapid beneficial effects on behavior in Smith-Lemli-Opitz syndrome and found no improvement in the ABC [28]. A prospective, double-blind, randomized, placebo-controlled, crossover trial of a phenylalanine-restricted diet performed in adults with late diagnosed phenylketonuria (PKU) and severe challenging behavior showed no differences in behavior as assessed by the ABC or the Vineland Adaptive Behavior Scales [29]. A randomized, double-blind, placebo-controlled, pilot trial investigating the effects of omega-3 fatty acids supplementation in autistic children exhibiting severe tantrums, aggression and self-injurious behavior failed to show between-group differences in the primary analysis, but did show improvement in the hyperactivity subscale in the treated cohort in a secondary analysis [30]. And a randomized, single-blind study of a gluten- and casein-free dietary intervention for children with ASD showed significant improvement in sub-domains of the ADOS (communication), Gilliam Autism Rating Scale (GARS) (social interaction) and Attention-Deficit Hyperactivity Disorder-IV (ADHD-IV) (inattention and hyperactivity) [31]. Our retrospective, exploratory study demonstrates a potential association between parent-reported use of soy-based infant formula and more severe autistic behaviors in sub-domains of the ABC (inappropriate speech, females) and ADI-R (RRSB, males) as well as several line-item scores of the ABC, ADI-R and ADOS Modules 1–3 in a population of high-functioning autistic children. Many of the potentially affected line-item scores relate to communication, which is also positively influenced by the gluten- and casein-free free dietary intervention in ASD [31].

Based on market sales, 12% of infant formulas in the United States are soy-based [17]. Approx 20–25% of infants receive some soy-based formula during their first year, but there is no data regarding how many are exclusively fed soy-based formula [18]. Parents may choose soy-based formula for their babies who are allergic to cow’s milk-based formula or because they themselves do not consume dairy products [18]. The primary ingredients in soy-based infant formulas are corn syrup, soy protein isolate, vegetable oils, sugar, vitamins and minerals. The soy protein isolate contains many toxic substances including saponins, protease inhibitors, phytic acid and phytoestrogens that can interfere with digestion, reproduction and thyroid function [18,31]. The National Toxicology Program (NTP)-Center for Evaluation of Risks to Human Reproduction (CERHR) reviewed the entire human and rodent literature regarding the safety of soy-based infant formulas and concluded that the evidence was insufficient to determine if soy infant formula was toxic at recommended intake levels in terms of bone mineral density, gastrointestinal effects, allergy/immunology, thyroid function, reproductive endpoints, cholesterol, diabetes mellitus and cognitive function. Thus, the evidence was also insufficient to determine if soy infant formula was safe at the recommended intake levels. None of the evaluated studies included subject populations genetically predisposed to developmental disorders such as ASD. These babies are more likely to have comorbid gastrointestinal and immunological issues that may precipitate the use of alternate formulas. The NTP-CERHR report did include 3 studies of infants with congenital hypothyroidism, which demonstrated that soy-based infant formula is associated with increased levels of thyroid-stimulating hormone (TSH) and thus complicates disease management [17]. Overall, there have been few longitudinal or disease-specific studies evaluating the effects of soy-based infant formula on development.

### Study design

The strength of this study design includes a large population of autistic children with comprehensive medical record histories and an autism diagnosis based on ADI-R and ADOS scores. Limitations of the study include: (1) data are retrospective regarding the type of formula, (2) there is low statistical power regarding the gender comparison analyses as there were significantly less female subjects than males, (3) there are no age-matched control data in typically developing children, and (4) the study population excluded autism subjects with a co-diagnosis of FXS and Down syndrome, which would be expected to be much lower functioning and perhaps more affected by environmental factors such as soy phytoestrogens. Despite these limitations, several autistic behaviors have been identified that are potentially associated with the consumption of soy-based infant formula (Table 11). These findings should be considered in the context of the exploratory nature of the analysis, as the SFARI data collection protocol was not specifically designed to assess the effects of infant formula on autistic behaviors nor powered to detect multiple hypotheses based on line-item analyses of diagnostic tests. A possible criticism is that sick children who were fed soy-based infant formula for various diagnosed or undiagnosed health complications were somehow predisposed to develop more severe autistic behaviors. Data regarding the reasons that subjects were fed soy-based infant formula, age at which soy formula was initiated and the length of time on soy-based infant formula are not available. A prospective study will be required to address these potentially confounding issues as well as longitudinal questions regarding the impact of soy-based infant formula on ASD throughout development.

In summary, the retrospective findings reported herein suggest that the use of soy-based infant formula may be associated with deficits in language, communication, social overtures and hypersensitivity to environmental stimuli in autistic children. These preliminary results raise important questions regarding the long-term effects of soy-based diets on autistic behaviors and deserve prospective investigation.

## Figures and Tables

**Table 1:** Aberrant behavior checklist scores dependent on soy formula.

	Soy		Non-Soy		
Males	Mean (N=297)	SD	Mean (N=1389)	SD	*P*
Total	48	25	46	26	0.20
Subscale 1	12	9.0	11	8.7	0.065
Subscale 2	10	7.0	9.6	7.2	0.23
Subscale 3	5.1	4.4	5.0	4.3	0.73
Subscale 4	17	10	17	11	0.40
Subscale 5	3.5	2.9	3.6	3.0	0.54
**Females**	**Mean (N=44)**	**SD**	**Mean (N=217)**	**SD**	**P**
Total	49	28	46	25	0.49
Subscale 1	12	9.3	12	8.3	0.77
Subscale 2	12	8.3	10	7.3	0.27
Subscale 3	5.4	4.7	4.7	4.4	0.34
Subscale 4	15	11	15	9.9	0.75
Subscale 5	4.4	3.2	3.4	2.9	0.049

**Table 2:** Line Item ABC scores dependent on soy-based formula use in autistic children.

ABC Question	Males				Females			
	Soy N=297	Non-Soy N=1389	Mean Change	P	Soy N=44	Non-Soy N=217	Mean Change	P
	Mean (SD)	Mean (SD)			Mean (SD)	Mean (SD)		
**Subscale 1: Irritability**								
2 injures self	0.29 (0.62)	0.23 (0.57)	0.06	0.16	0.32 (0.67)	0.24 (0.54)	0.08	0.43
4 aggressive toward others	0.79 (0.89)	0.75 (0.90)	0.04	0.42	0.57 (0.79)	0.67 (0.83)	−0.10	0.46
8 screams inappropriately	0.89 (0.96)	0.85 (0.96)	0.04	0.51	0.84 (1.1)	0.98 (0.97)	−0.14	0.39
10 temper tantrums/outbursts	1.3 (0.97)	1.2 (0.96)	0.1	0.17	1.1 (0.93)	1.3 (0.97)	−0.2	0.33
14 irritable and whiny	0.94 (0.91)	0.91 (0.90)	0.03	0.66	0.95 (0.86)	1.0 (0.89)	−0.05	0.57
19 yells at inappropriate times	0.94 (0.97)	0.89 (0.96)	0.05	0.43	0.91 (1.0)	0.94 (0.92)	−0.03	0.86
25 depressed mood	0.35 (0.67)	0.28 (0.61)	0.07	0.10	0.43 (0.85)	0.35 (0.64)	0.08	0.50
29 demands must be met immediately	1.3 (1.0)	1.2 (1.0)	0.1	0.072	1.2 (1.0)	1.3 (1.0)	−0.1	0.88
34 cries over minor hurts	0.93 (1.0)	0.80 (0.94)	0.13	0.030	1.2 (1.1)	0.86 (0.94)	0.34	0.059
36 mood changes quickly	0.97 (0.92)	0.87 (0.93)	0.10	0.11	1.3 (1.1)	1.0 (0.96)	0.2	0.15
41 cries/screams inappropriately	0.76 (0.93)	0.72 (0.94)	0.04	0.48	0.70 (0.95)	0.91 (1.0)	−0.21	0.21
47 stamps feet/bangs objects/slams doors	0.81 (1.0)	0.71 (0.93)	0.10	0.089	0.82 (1.0)	0.72 (0.95)	0.10	0.55
50 deliberately hurts him/herself	0.28 (0.64)	0.23 (0.58)	0.05	0.22	0.36 (0.78)	0.24 (0.53)	0.12	0.18
52 physical violence to self	0.22 (0.56)	0.20 (0.54)	0.02	0.48	0.34 (0.75)	0.18 (0.50)	0.16	0.087
57 temper outbursts if not own way	1.5 (1.0)	1.3 (1.0)	0.2	0.077	1.5 (0.93)	1.4 (1.0)	0.1	0.65
**Subscale 2: Lethargy**								
3 listless, sluggish, inactive	0.43 (0.73)	0.35 (0.66)	0.08	0.062	0.36 (0.78)	0.44 (0.74)	−0.08	0.55
5 seeks isolation from others	1.0 (0.86)	1.0 (0.89)	0	0.38	1.0 (1.0)	1.1 (0.89)	−0.1	0.58
12 preoccupied, stares into space	0.89 (0.81)	0.88 (0.85)	0.01	0.85	0.98 (0.90)	0.85 (0.82)	0.13	0.37
16 withdrawn, prefers solitary activities	1.2 (0.96)	1.1 (0.94)	0.1	0.15	1.4 (1.1)	1.2 (0.97)	0.2	0.41
20 fixed facial expression	0.55 (0.78)	0.48 (0.72)	0.07	0.13	0.52 (0.85)	0.58 (0.80)	−0.06	0.69
23 does nothing but sit and watch others	0.24 (0.52)	0.23 (0.52)	0.01	0.78	0.39 (0.69)	0.24 (0.59)	0.15	0.16
26 resists any form of physical contact	0.24 (0.55)	0.25 (0.53)	−0.01	0.65	0.36 (0.61)	0.33 (0.62)	0.03	0.75
30 isolates him/herself	0.95 (0.84)	0.87 (0.86)	0.08	0.15	1.2 (0.98)	0.94 (0.86)	0.26	0.074
32 sits/stands in one position for a long time	0.23 (0.58)	0.24 (0.58)	−0.01	0.91	0.36 (0.87)	0.26 (0.59)	0.10	0.35
37 unresponsive to structured activities	0.53 (0.76)	0.52 (0.77)	0.01	0.87	0.60 (0.93)	0.50 (0.79)	0.10	0.45
40 is difficult to reach/contact/get through to	1.0 (0.88)	0.95 (0.87)	0.05	0.45	1.0 (1.0)	0.97 (0.87)	0.03	0.71
42 prefers to be alone	1.0 (0.90)	0.95 (0.93)	0.05	0.29	1.4 (1.1)	1.0 (0.94)	0.4	0.030
43 does not try to communicate	0.43 (0.81)	0.39 (0.75)	0.04	0.41	0.70 (0.93)	0.47 (0.81)	0.23	0.088
53 inactive, no spontaneous movement	0.12 (0.42)	0.10 (0.37)	0.02	0.30	0.14 (0.63)	0.12 (0.35)	0.02	0.81
55 responds negatively to affection	0.27 (0.59)	0.27 (0.57)	0	0.98	0.41 (0.66)	0.33 (0.63)	0.08	0.44
58 shows few social relations to others	1.0 (0.92)	0.99 (0.90)	0.01	0.90	1.0 (0.90)	1.1 (1.0)	−0.1	0.75
**Subscale 3: Stereotypy**								
6 meaningless, recurrent body movements	0.98 (0.96)	0.96 (0.95)	0.02	0.71	0.84 (1.1)	0.91 (0.99)	−0.07	0.66
11 stereotypical behavior	1.1 (0.97)	1.1 (0.98)	0	0.88	1.2 (1.0)	0.97 (0.98)	0.23	0.20
17 odd, bizarre behavior	0.96 (0.90)	0.99 (0.90)	−0.03	0.64	0.98 (0.90)	1.0 (0.92)	−0.02	0.76
27 move or rolls head back/forth repeatedly	0.16 (0.48)	0.15 (0.46)	0.01	0.61	0.25 (0.65)	0.12 (0.46)	0.13	0.13
35 repetitive hand/body/head movements	0.90 (0.99)	0.91 (0.97)	−0.01	0.95	0.98 (1.0)	0.85 (0.97)	0.13	0.44
45 waves or shakes extremities repeatedly	0.67 (0.93)	0.62 (0.89)	0.05	0.42	0.80 (0.90)	0.54 (0.84)	0.26	0.069
49 repeatedly rocks back and forth	0.29 (0.65)	0.25 (0.60)	0.04	0.35	0.36 (0.87)	0.26 (0.61)	0.10	0.34
**Subscale 4: Hyperactivity**								
1 excessively active	1.1 (0.99)	1.0 (1.0)	0.1	0.31	0.82 (0.95)	0.90 (0.97)	−0.08	0.61
7 boisterous	1.0 (0.94)	1.0 (0.95)	0	0.60	0.86 (1.0)	0.82 (0.91)	0.04	0.80
13 impulsive	1.4 (1.0)	1.3 (0.99)	0.1	0.11	1.0 (1.0)	1.2 (0.94)	−0.2	0.18
15 restless, unable to sit still	1.3 (1.0)	1.3 (1.0)	0	0.35	1.1 (1.1)	1.1 (0.99)	0	0.76
18 disobedient, difficult to control	1.0 (0.93)	0.98 (0.96)	0.02	0.62	0.82 (0.92)	0.93 (0.87)	−0.11	0.46
21 disturbs others	1.1 (0.91)	1.1 (0.92)	0	0.12	0.73 (0.85)	0.98 (0.90)	−0.25	0.091
24 uncooperative	0.81 (0.84)	0.85 (0.85)	−0.04	0.44	0.61 (0.78)	0.90 (0.79)	−0.29	0.030
28 does not pay attention to instructions	1.3 (0.82)	1.3 (0.86)	0	0.82	1.3 (1.0)	1.2 (0.81)	0.1	0.84
31 disrupts group activities	0.95 (0.95)	0.95 (0.91)	0	0.95	0.95 (1.0)	0.87 (0.91)	0.08	0.56
38 does not stay in seat	1.2 (0.99)	1.1 (1.0)	0.1	0.33	1.1 (1.2)	1.0 (1.0)	0.1	0.71
39 will not sit still for any length of time	0.94 (0.93)	0.93 (0.96)	0.01	0.83	0.82 (1.1)	0.84 (0.95)	−0.02	0.88
44 easily distracted	1.5 (0.99)	1.5 (0.99)	0	0.98	1.4 (1.0)	1.4 (0.98)	0	0.89
48 constantly runs or jumps	0.96 (1.0)	0.92 (1.0)	0.04	0.53	0.91 (1.0)	0.78 (1.0)	0.13	0.45
51 pays no attention when spoken to	0.96 (0.82)	0.97 (0.79)	−0.01	0.87	0.93 (0.90)	1.0 (0.84)	−0.07	0.63
54 tends to be excessively active	1.1 (1.1)	0.97 (1.1)	0.13	0.24	0.91 (1.1)	0.76 (0.98)	0.15	0.38
56 deliberately ignores directions	0.81 (0.85)	0.74 (0.85)	0.07	0.20	0.70 (0.95)	0.67 (0.75)	0.03	0.81
**Subscale 5: Inappropriate Speech**								
9 talks excessively	0.86 (0.99)	0.77 (0.94)	0.09	0.13	0.86 (0.98)	0.68 (0.91)	0.18	0.22
22 repetitive speech	1.1 (0.99)	1.2 (1.0)	−0.1	0.12	1.4 (1.1)	1.1 (0.97)	0.3	0.10
33 talks to self loudly	0.61 (0.84)	0.65 (0.89)	−0.04	0.49	0.89 (1.0)	0.64 (0.93)	0.25	0.12
46 repeats a word or phrase over and over	0.99 (1.1)	1.1 (1.0)	−0.11	0.32	1.3 (1.1)	1.0 (1.0)	0.3	0.11

**Table 3:** ADI-R scores dependent on soy-based formula.

	Soy			Non Soy			
Males	N	Mean	SD	N	Mean	SD	*P*
Total (RSI+NV^a^+RRSB)	297	37	8.9	1390	36	9.3	0.23
Total RSI^b^	297	21	5.7	1390	20	5.8	0.34
Total Verbal	257	16	4.5	1210	16	4.2	0.41
Total Non Verbal	297	9.2	3.4	1390	9.3	3.4	0.54
Total RRSB^c^	297	6.9	2.4	1390	6.4	2.5	<0.01
**Females**	**N**	**Mean**	**SD**	**N**	**Mean**	**SD**	***P***
Total (RSI+NV^a^+RRSB)	44	37	9.7	217	36	10	0.80
Total RSI^b^	44	21	5.3	217	21	6.1	0.70
Total Verbal	36	17	4.4	190	17	4.3	0.84
Total Non Verbal	44	9.3	4.0	217	9.7	3.5	0.52
Total RRSB^c^	44	6.3	2.2	217	5.9	2.4	0.28

a Non Verbal

b Reciprocol Social Interaction (RSI)

c Restricted, Repetitive and Stereotyped Behavior (RRSB)

**Table 4:** ADI-R sub-scores dependent on soy-based formula.

	Soy (N=297)		Non Soy (N=1391)		
Males	Mean	SD	Mean	SD	*P*
A1^a^ failure nonverbal social	3.8	1.6	3.8	1.7	0.81
A2 failure peer relationships	6.1	1.7	6.0	1.8	0.70
A3 lack shared enjoy	4.4	1.6	4.2	1.7	0.15
A4 lack socioemotional	6.2	2.2	6.1	2.2	0.21
B1^b^ delay language gestures	4.5	2.7	4.7	2.6	0.34
B2 fail initiate conversation	3.6	0.82	3.6	0.68	0.18
B3 idiosyncratic speech	4.0	1.8	3.9	1.8	0.72
B4 lack make believe	4.7	1.3	4.6	1.3	0.72
C1^c^ encompassing pattern	2.1	1.2	1.9	1.2	<0.01
C2 compulsive rituals	1.6	1.4	1.5	1.4	0.19
C3 repetitive mannerisms	1.5	0.77	1.4	0.80	0.26
C4 preoccupation material	1.7	0.53	1.7	0.60	0.16
	**Soy (N=44)**		**Non Soy (N=217)**		
**Females**	**Mean**	**SD**	**Mean**	**SD**	***P***
A1^a^ failure nonverbal social	3.9	1.3	4.0	1.7	0.67
A2 failure peer relationships	6.3	1.8	6.0	1.7	0.48
A3 lack shared enjoy	4.4	1.9	4.4	1.8	0.87
A4 lack socioemotional	6.4	2.1	6.2	2.3	0.53
B1^b^ delay language gestures	4.7	3.0	5.1	2.6	0.29
B2 fail initiate conversation	3.8	0.45	3.7	0.62	0.28
B3 idiosyncratic speech	4.5	1.5	3.9	1.8	0.10
B4 lack make believe	4.6	1.5	4.5	1.4	0.74
C1^c^ encompassing pattern	1.6	1.0	1.5	1.1	0.32
C2 compulsive rituals	1.6	1.5	1.4	1.4	0.41
C3 repetitive mannerisms	1.4	0.82	1.4	0.81	0.98
C4 preoccupation material	1.6	0.57	1.6	0.66	0.63

a A1–A4 denote RSI sub-categories

b B1–B4 denote Non Verbal sub-categories

c C1–C4 denote RRSB sub-categories

**Table 5:** ADI-R individual question scores dependent on soy-based formula.

	Males					Females				
	Soy (N=286)		Non Soy (N=1332)			Soy (N=43)		Non Soy (N=201)		
	Mean	SD	Mean	SD	P*	Mean	SD	Mean	SD	P
13. Loss of spontaneous use of at least 5 meaningful words	0.32	0.72	0.26	0.67	0.18	0.28	0.67	0.22	0.76	0.57
14. Loss of communicative intent	0.31	0.72	0.22	0.61	0.027	0.33	0.75	0.14	0.67	0.045
15. Loss of syntactical skills (grammar)	0.028	0.24	0.019	0.19	0.48	0	0	0.01	0.21	0.64
16. Loss of articulation (pronunciation)	0.077	0.39	0.051	0.31	0.22	0.07	0.34	0.030	0.29	0.37
20. Loss of skills for at least 3 months	0.54	0.86	0.47	0.83	0.22	0.40	0.79	0.48	0.88	0.53
23. Self-help skills (feeding, dress, using bathroom etc)	0.084	0.37	0.065	0.34	0.38	0.047	0.30	0.040	0.27	0.88
27. Assoc of loss of skills with physical illness	0.077	0.32	0.065	0.27	0.52	0.023	0.15	0.055	0.20	0.46
29. Comprehensive of simple language-most abnormal	1.4	0.86	1.3	0.89	0.023	1.4	0.77	1.4	0.79	0.84
30. Overall level of language	0.19	0.51	0.17	0.48	0.60	0.26	0.58	0.15	0.47	0.17
31. Use of other	0.88	0.90	0.81	0.86	0.21	0.91	0.92	0.82	0.92	0.57
32. Articulation/pronunciation-at 5.0 years	0.57	0.83	0.61	0.85	0.45	0.56	0.85	0.60	0.87	0.79
34. Social verbalization/chat-current	0.94	0.78	1.0	0.73	0.059	0.93	0.77	1.1	0.72	0.16
34. Social verbalization/chat-ever	1.5	0.79	1.6	0.74	0.44	1.5	0.83	1.6	0.76	0.45
36. Inappropriate questions or statements-current	0.74	0.81	0.69	0.80	0.34	0.53	0.70	0.71	0.80	0.19
36. Inappropriate questions or statements-ever	0.86	0.86	0.80	0.85	0.25	0.67	0.87	0.81	0.84	0.36
37. Pronominal reversal-current	0.45	0.76	0.44	0.74	0.89	0.60	0.90	0.57	0.84	0.80
37. Pronominal reversal-ever	0.88	0.91	0.89	0.90	0.84	1.0	0.98	0.96	0.91	0.80
39. Verbal rituals-current	0.56	0.82	0.52	0.80	0.43	0.60	0.88	0.54	0.77	0.61
39. Verbal rituals-ever	0.65	0.87	0.63	0.86	0.72	0.67	0.92	0.64	0.80	0.80
41. Current communicative speech-at 5.0 years	0.65	0.68	0.67	0.70	0.71	0.63	0.66	0.72	0.76	0.45
42. Pointing to express interest-most abnormal	1.4	0.74	1.3	0.75	0.25	1.3	0.77	1.4	0.72	0.17
43. Nodding-current	0.69	0.87	0.69	0.85	0.97	0.67	0.89	0.84	0.91	0.29
44. Head shaking-most abnormal	0.74	0.90	0.86	0.90	0.041	0.86	0.97	1.1	0.92	0.19
44. Head shaking-current	0.51	0.78	0.59	0.80	0.13	0.56	0.85	0.75	0.90	0.21
45. Conventional/instrumental gestures-most abnormal	1.4	0.80	1.4	0.77	0.90	1.3	0.87	1.5	0.72	0.32
45. Conventional/instrumental gestures-current	0.97	0.85	0.98	0.80	0.90	0.95	0.92	1.0	0.82	0.61
46. Attention to voice-current	0.19	0.53	0.16	0.49	0.44	0.23	0.57	0.17	0.51	0.48
47. Spontaneous imitation of actions-current	0.92	0.93	0.97	0.91	0.41	0.98	0.86	0.88	0.89	0.51
50. Direct gaze-most abnormal	1.4	0.64	1.4	0.66	0.47	1.2	0.57	1.5	0.62	0.016
50. Direct gaze-current	0.14	0.43	0.14	0.41	0.84	0.14	0.35	0.16	0.52	0.79
51. Social smiling-most abnormal	1.3	0.78	1.3	0.76	0.45	1.3	0.71	1.2	0.78	0.45
52. Showing and directing attention-most abnormal	1.4	0.78	1.4	0.81	0.15	1.5	0.77	1.4	0.79	0.59
52. Showing and directing attention-current	0.92	0.87	0.80	0.84	0.025	0.86	0.89	0.86	0.90	1.0
54. Seeking to share her/his enjoyment with others-most abnormal	1.2	0.76	1.2	0.79	0.40	1.2	0.85	1.2	0.80	0.73
54. Seeking to share her/his enjoyment with others-current	0.62	0.68	0.61	0.70	0.82	0.72	0.73	0.69	0.77	0.81
55. Offering comfort-most abnormal	1.4	0.74	1.4	0.74	0.97	1.5	0.70	1.4	0.67	0.64
55. Offering comfort-current	0.86	0.76	0.83	0.75	0.49	1.1	0.71	0.89	0.72	0.040
56. Quality of social overture-most abnormal	1.3	0.71	1.3	0.74	0.081	1.3	0.69	1.3	0.70	0.62
56. Quality of social overture-current	0.79	0.73	0.69	0.69	0.029	0.79	0.80	0.71	0.67	0.45
57. Range of facial expressions used to communicate-most abnormal	1.2	0.75	1.2	0.74	0.96	1.3	0.67	1.3	0.69	0.84
57. Range of facial expressions used to communicate-current	0.79	0.66	0.79	0.64	0.97	0.84	0.61	0.89	0.63	0.66
58. Inappropriate facial expressions-current	1.1	0.76	0.95	0.76	0.047	0.86	0.71	0.94	0.78	0.54
58. Inappropriate facial expressions-ever	1.2	0.77	1.1	0.78	0.15	1.1	0.66	1.1	0.80	0.93
59. Appropriateness of social response-most abnormal	1.5	0.62	1.5	0.60	0.87	1.5	0.59	1.5	0.60	0.79
61. Imitative social play-most abnormal	1.3	0.70	1.3	0.69	0.58	1.2	0.68	1.2	0.63	0.92
63. Response to approaches of other children-most abnormal	1.2	0.61	1.2	0.65	0.61	1.3	0.68	1.2	0.64	0.31
63. Response to approaches of other children-current	0.54	0.64	0.52	0.62	0.57	0.74	0.69	0.61	0.70	0.23
65. Friendships-current	1.3	0.80	1.3	0.84	0.27	1.3	0.89	1.3	0.80	0.79
67. Unusual preoccupations-current	0.48	0.77	0.38	0.72	0.048	0.30	0.67	0.27	0.62	0.72
70. Compulsions/rituals-current	0.80	0.90	0.68	0.87	0.024	0.81	0.96	0.69	0.86	0.39
70. Compulsions/rituals-ever	0.94	0.93	0.83	0.92	0.082	0.98	0.99	0.80	0.94	0.26
71. Unusual sensory interests-current	0.97	0.71	0.85	0.70	0.016	0.93	0.74	0.85	0.72	0.50
71. Unusual sensory interests -ever	1.2	0.70	1.1	0.74	0.12	1.2	0.73	1.1	0.76	0.47
72. Undue general sensitivity to noise-current	1.2	0.88	1.1	0.91	0.033	0.91	0.89	1.1	0.93	0.35
72. Undue general sensitivity to noise-ever	1.5	0.83	1.4	0.86	0.28	1.5	0.80	1.3	0.88	0.18
73. Abnormal, idiosyn, negative response to spec sensory stim-current	0.92	0.85	0.81	0.83	0.043	0.63	0.85	0.81	0.85	0.21
73. Abnormal, idiosyn, negative response to spec sensory stim-ever	1.1	0.87	0.98	0.89	0.14	0.95	0.95	1.0	0.88	0.62
74. Difficulties with minor changes routines/personal env-current	1.1	0.83	0.97	0.83	0.024	0.79	0.89	0.96	0.83	0.26
74. Difficulties with minor changes routines/personal env-ever	1.3	0.84	1.2	0.86	0.020	1.0	0.96	1.2	0.90	0.37
75. Resistance to trivial changes in environment-current	0.23	0.50	0.23	0.54	0.96	0.12	0.39	0.24	0.63	0.20
76. Unusual attachment to objects-current	0.56	1.4	0.45	1.2	0.16	0.26	0.54	0.39	1.2	0.43
77. Hand and finger mannerisms-current	0.85	0.88	0.83	0.88	0.79	0.93	0.94	0.82	0.93	0.47
78. Other complex mannerisms or stereotyped body movements-current	0.93	0.92	0.74	0.87	<0.01	0.77	0.90	0.79	0.90	0.88
79. Midline hand movements-current	0.059	0.29	0.063	0.29	0.84	0.070	0.26	0.070	0.25	1.0
81. Aggression toward caregivers or family members-ever	1.3	0.85	1.2	0.85	0.062	1.2	0.92	1.2	0.92	0.83
82. Aggression toward noncaregivers or nonfamily members-ever	0.84	0.88	0.89	0.90	0.41	0.79	0.94	0.70	0.92	0.56
83. Self-injury-current	0.49	0.70	0.38	0.64	<0.01	0.53	0.77	0.47	0.68	0.57
83. Self-injury-ever	0.76	0.81	0.67	0.80	0.086	0.91	0.89	0.72	0.83	0.18
84. Hyperventilation-current	0.080	0.32	0.068	0.28	0.49	0.070	0.26	0.055	0.18	0.71
84. Hyperventilation-ever	0.12	0.41	0.10	0.35	0.42	0.093	0.37	0.080	0.23	0.80
86. Age when abnormality first evident	3.4	0.94	3.4	0.94	0.78	3.3	0.98	3.4	0.79	0.42
88. Visiospatial skill-current	0.17	0.46	0.19	0.50	0.44	0.17	0.44	0.13	0.42	0.57
88. Visiospatial skill-ever	0.22	0.54	0.25	0.57	0.55	0.21	0.52	0.19	0.45	0.78
89. Memory skill-current	0.55	0.77	0.47	0.75	0.11	0.72	0.83	0.49	0.69	0.064
89. Memory skill-ever	0.56	0.78	0.49	0.76	0.16	0.70	0.83	0.51	0.70	0.15
91. Drawing skill-current	0.091	0.37	0.073	0.33	0.41	0.093	0.37	0.12	0.28	0.72
92. Reading ability-current	0.17	0.52	0.21	0.55	0.29	0.16	0.48	0.22	0.53	0.54
92. Reading ability-ever	0.26	0.63	0.28	0.63	0.62	0.23	0.61	0.30	0.62	0.57

**Table 6:** ADOS scores dependent on soy-based formula.

Males	Soy (N=291)		Non Soy (N=1362)		
	Mean	SD	Mean	SD	*P*
Total	28	8.9	28	9.0	0.67
Total CSI^a^	13	4.0	13	4.1	0.48
Total RRB^b^	4.0	2.1	4.0	2.0	0.50
Total Social Affect	11	3.9	11	3.9	0.91
**Females**	**Soy (N=44)**		**Non Soy (N=215)**		
	**Mean**	**SD**	**Mean**	**SD**	***P***
Total	29	8.5	29	9.8	0.73
Total CSI^a^	13	3.8	14	4.5	0.58
Total RRB^b^	4.2	2.2	4.1	2.2	0.81
Total Social Affect	11	3.9	11	4.1	0.74

a Communication & Social Interaction (CSI)

b Restricted & Repetitive Behavior (RRB)

**Table 7:** ADOS module 1 sub-scores dependent on soy-based formula.

Males	Soy (N=62)		Non Soy (N=257)		
	Mean	SD	Mean	SD	P*
A1 overall level nonechoed language	1.3	0.80	1.2	0.79	0.30
A2 frequency vocalization directed to others	1.5	0.59	1.3	0.54	0.041
A3 intonation of vocalization or verbalizations	1.5	0.80	1.7	0.66	0.082
A4 immediate echolalia	0.73	0.79	0.78	0.76	0.63
A5 stereotyped/idiosyncratic use of words/phrases	0.47	0.80	0.66	0.87	0.12
A6 use of other	0.77	0.93	0.67	0.89	0.43
A7 pointing	1.5	0.59	1.3	0.67	0.022
A8 gestures	1.4	0.67	1.3	0.74	0.25
B1 unusual eye contact	2.0	0.25	2.0	0.25	0.97
B2 responsive social smile	1.3	0.79	1.3	0.79	0.54
B3 facial expressions directed to others	1.2	0.43	1.3	0.54	0.57
B4 integration of gaze and other behaviors	1.4	0.64	1.2	0.65	0.072
B5 shared enjoyment in interaction	0.89	0.70	1.0	0.73	0.23
B6 response to name	0.81	0.83	0.86	0.81	0.62
B7 requesting	0.90	0.56	0.65	0.64	<0.01
B8 giving	1.4	0.58	1.3	0.59	0.77
B9 showing	1.7	0.46	1.8	0.49	0.44
B10 spontaneous initiation of joint attention	1.8	0.53	1.6	0.67	0.052
B11 response to joint attention	0.69	0.76	0.46	0.70	0.020
B12 quality of social overtures	1.7	0.47	1.7	0.51	0.78
C1 functional play with objects	1.6	0.64	1.5	0.68	0.44
C2 imagination/creativity	1.7	0.56	1.6	0.60	0.54
D1 unusual sensory interest in play material/person	1.4	0.74	1.4	0.77	0.70
D2 hand and finger and other complex mannerisms	1.5	0.82	1.3	0.87	0.29
D3 self-injurious behavior	0.18	0.50	0.20	0.53	0.78
D4 unusually repetitive interests or stereotyped behaviors	1.4	0.69	1.5	0.65	0.61
E1 overactivity	0.5	0.65	0.42	0.65	0.41
E2 tantrums, aggression, negative or disruptive behavior	0.42	0.59	0.34	0.60	0.36
E3 anxiety	0.097	0.30	0.066	0.26	0.43
**Females**	**Soy (N=14)**		**Non Soy (N=47)**		
	**Mean**	**SD**	**Mean**	**SD**	***P***
A1 overall level nonechoed language	1.0	0.68	1.4	0.71	0.078
A2 frequency vocalization directed to others	1.3	0.73	1.3	0.58	0.96
A3 intonation of vocalization or verbalizations	1.6	0.63	1.7	0.57	0.57
A4 immediate echolalia	0.71	0.91	0.96	0.78	0.33
A5 stereotyped/idiosyncratic use of words/phrases	1.1	0.95	0.79	0.93	0.22
A6 use of other	0.29	0.73	0.62	0.87	0.20
A7 pointing	1.2	0.70	1.5	0.66	0.15
A8 gestures	1.1	0.86	1.4	0.67	0.32
B1 unusual eye contact	2.0	0	2.0	0.29	0.59
B2 responsive social smile	1.4	0.76	1.4	0.68	0.91
B3 facial expressions directed to others	1.3	0.47	1.4	0.50	0.29
B4 integration of gaze and other behaviors	1.1	0.62	1.3	0.67	0.37
B5 shared enjoyment in interaction	0.93	0.83	1.1	0.74	0.39
B6 response to name	0.79	0.89	0.89	0.84	0.68
B7 requesting	0.43	0.65	0.72	0.62	0.12
B8 giving	1.6	0.51	1.2	0.56	0.049
B9 showing	1.6	0.63	1.8	0.38	0.18
B10 spontaneous initiation of joint attention	1.1	0.86	1.7	0.70	0.025
B11 response to joint attention	0.50	0.76	0.43	0.68	0.73
B12 quality of social overtures	1.6	0.51	1.6	0.49	0.66
C1 functional play with objects	1.5	0.52	1.3	0.74	0.25
C2 imagination/creativity	1.2	0.70	1.5	0.72	0.21
D1 unusual sensory interest in play material/person	1.2	0.58	1.4	0.77	0.40
D2 hand and finger and other complex mannerisms	1.3	0.99	1.2	0.93	0.80
D3 self-injurious behavior	0	0	0.064	0.25	0.34
D4 unusually repetitive interests or stereotyped behaviors	1.3	0.83	1.5	0.65	0.25
E1 overactivity	0.14	0.36	0.28	0.58	0.42
E2 tantrums, aggression, negative or disruptive behavior	0.21	0.43	0.47	0.62	0.16
E3 anxiety	0.14	0.36	0.085	0.28	0.53

* Student t-test

**Table 8:** ADOS module 2 sub-scores dependent on soy-based formula.

Males	Soy (N=57)		Non Soy (N=308)		
	Mean	SD	Mean	SD	*P*
A1 overall level nonechoed language	0.47	0.63	0.43	0.59	0.60
A2 amount of social overtures/maintenance of attention	0.98	0.72	1.0	0.71	0.67
A3 speech abnormalities associated with autism	1.6	0.62	1.5	0.63	0.36
A4 immediate echolalia	0.96	0.78	0.94	0.77	0.81
A5 stereotyped/idiosyncratic use of words/phrases	1.4	0.68	1.4	0.69	0.71
A6 conversation	1.7	0.57	1.7	0.52	0.80
A7 pointing	1.0	0.67	0.94	0.65	0.38
A8 descriptive, conventional, instrumental or informal gestures	1.0	0.69	1.1	0.69	0.35
B1 unusual eye contact	1.9	0.37	1.9	0.44	0.59
B2 facial expression directed to others	1.1	0.55	1.0	0.52	0.86
B3 shared enjoyment in interaction	0.65	0.72	0.67	0.72	0.87
B4 respond to name	0.47	0.68	0.41	0.63	0.51
B5 showing	1.2	0.71	1.2	0.74	0.40
B6 spontaneous initiation of joint attention	1.3	0.78	1.3	0.77	0.72
B7 response to joint attention	0.14	0.35	0.091	0.33	0.30
B8 quality of social overtures	1.4	0.53	1.2	0.59	0.049
B9 quality of social response	1.4	0.55	1.4	0.56	0.81
B10 reciprocal social communication	1.2	0.73	1.3	0.71	0.16
B11 overall quality of rapport	1.2	0.71	1.4	0.67	0.17
C1 function play with objects	0.63	0.77	0.53	0.69	0.33
C2 imagination/creativity	1.2	0.66	1.0	0.70	0.037
D1 unusual sensory interest in play material/person	1.2	0.79	1.3	0.79	0.56
D2 hand and finger and other complex mannerisms	1.0	0.89	0.88	0.89	0.35
D3 self-injurious behavior	0.12	0.47	0.058	0.31	0.19
D4 unusually repetitive interests or stereotyped behaviors	1.3	0.58	1.3	0.65	0.84
E1 overactivity	0.40	0.59	0.35	0.54	0.50
E2 tantrums, aggression, negative or disruptive behavior	0.23	0.50	0.28	0.53	0.53
E3 anxiety	0.11	0.36	0.091	0.30	0.75
**Females**	**Soy (N=11)**		**Non Soy (N=57)**		
	**Mean**	**SD**	**Mean**	**SD**	***P***
A1 overall level nonechoed language	0.72	0.79	0.61	0.67	0.62
A2 amount of social overtures/maintenance of attention	0.73	0.79	1.2	0.79	0.049
A3 speech abnormalities associated with autism	1.7	0.47	1.6	0.62	0.63
A4 immediate echolalia	1.0	0.77	1.3	0.78	0.25
A5 stereotyped/idiosyncratic use of words/phrases	1.5	0.69	1.5	0.66	0.87
A6 conversation	1.8	0.40	1.8	0.41	0.83
A7 pointing	0.54	0.52	1.1	0.59	<0.01
A8 descriptive, conventional, instrumental or informal gestures	0.73	0.79	1.3	0.60	<0.01
B1 unusual eye contact	2.0	0	1.9	0.37	0.54
B2 facial expression directed to others	1.2	0.40	1.2	0.50	0.97
B3 shared enjoyment in interaction	0.45	0.69	0.80	0.77	0.16
B4 respond to name	0.1	0.32	0.60	0.78	0.051
B5 showing	1.0	0.89	1.2	0.79	0.36
B6 spontaneous initiation of joint attention	0.82	0.87	1.4	0.70	0.025
B7 response to joint attention	0	0	0.12	0.38	0.29
B8 quality of social overtures	1.3	0.65	1.4	0.59	0.63
B9 quality of social response	1.4	0.50	1.5	0.63	0.53
B10 reciprocal social communication	1.3	0.65	1.5	0.66	0.24
B11 overall quality of rapport	0.82	0.60	1.4	0.68	<0.01
C1 function play with objects	0.27	0.65	0.47	0.66	0.36
C2 imagination/creativity	0.64	0.81	0.86	0.55	0.26
D1 unusual sensory interest in play material/person	1.3	0.79	1.3	0.79	0.82
D2 hand and finger and other complex mannerisms	1.1	0.70	0.98	0.94	0.72
D3 self-injurious behavior	0.18	0.60	0.088	0.39	0.51
D4 unusually repetitive interests or stereotyped behaviors	1.1	0.54	1.4	0.72	0.26
E1 overactivity	0.27	0.47	0.39	0.56	0.53
E2 tantrums, aggression, negative or disruptive behavior	0.36	0.50	0.32	0.60	0.81
E3 anxiety	0.27	0.47	0.035	0.19	<0.01

**Table 9:** ADOS module 3 sub-scores dependent on soy-based formula.

Males	Soy (N=172)		Non Soy (N=797)		
	Mean	SD	Mean	SD	*P*
A1 overall level nonechoed language	0.40	0.49	0.40	0.49	0.91
A2 speech abnormalities associated with autism	1.4	0.68	1.4	0.63	0.75
A3 immediate echolalia	0.18	0.46	0.20	0.44	0.56
A4 stereotyped/idiosyncratic use of words/phrases	1.2	0.65	1.2	0.66	0.62
A5 offers information	0.28	0.54	0.44	0.65	<0.01
A6 asks for information	0.92	0.72	1.1	0.76	0.024
A7 reporting of events	0.62	0.63	0.74	0.71	0.046
A8 conversation	1.0	0.59	1.1	0.65	0.47
A9 descriptive, conventional, instrumental or inform gestures	0.56	0.58	0.53	0.63	0.62
B1 unusual eye contact	1.8	0.55	1.8	0.64	0.18
B2 facial expression directed to others	0.98	0.51	0.97	0.54	0.84
B3 language production and linked non verbal comm	0.087	0.32	0.079	0.27	0.73
B4 shared enjoyment in interaction	0.66	0.69	0.61	0.72	0.48
B5 empathy/comments on others	1.3	0.68	1.3	0.71	0.30
B6 insight	1.5	0.63	1.6	0.56	0.025
B7 quality of social overtures	1.0	0.51	1.1	0.48	0.10
B8 quality of social response	1.1	0.44	1.1	0.50	0.78
B9 amount of reciprocal social communication	0.66	0.68	0.75	0.69	0.14
B10 overall quality of rapport	0.95	0.66	1.1	0.59	0.055
C1 imagination/creativity	0.80	0.66	0.84	0.70	0.50
D1 unusual sensory interest in play material/person	0.73	0.84	0.70	0.78	0.63
D2 hand and finger and other complex mannerisms	0.36	0.63	0.46	0.74	0.10
D3 self-injurious behavior	0.058	0.26	0.055	0.24	0.89
D4 excs inter/ref to unus/highly spec topics/objts/rep behav	0.90	0.77	0.96	0.78	0.35
D5 compulsions or rituals	0.45	0.63	0.51	0.66	0.31
E1 overactivity/agitation	0.41	0.63	0.36	0.57	0.30
E2 tantrums, aggression, negative or disruptive behavior	0.087	0.32	0.13	0.37	0.15
E3 anxiety	0.13	0.35	0.11	0.34	0.48
**Females**	**Soy (N=19)**		**Non Soy (N=111)**		
	**Mean**	**SD**	**Mean**	**SD**	***P***
A1 overall level nonechoed language	0.53	0.51	0.37	0.48	0.20
A2 speech abnormalities associated with autism	1.4	0.68	1.3	0.65	0.79
A3 immediate echolalia	0.11	0.32	0.17	0.44	0.54
A4 stereotyped/idiosyncratic use of words/phrases	1.0	0.82	1.1	0.64	0.67
A5 offers information	0.68	0.82	0.32	0.57	0.020
A6 asks for information	1.2	0.83	1.1	0.81	0.70
A7 reporting of events	0.84	0.69	0.58	0.65	0.11
A8 conversation	1.1	0.66	1.1	0.58	0.82
A9 descriptive, conventional, instrumental or inform gestures	0.89	0.74	0.56	0.64	0.041
B1 unusual eye contact	1.8	0.63	1.7	0.69	0.72
B2 facial expression directed to others	1.0	0.47	0.89	0.51	0.39
B3 language production and linked non verbal comm	0.11	0.32	0.045	0.21	0.29
B4 shared enjoyment in interaction	0.63	0.68	0.44	0.57	0.19
B5 empathy/comments on others	1.2	0.76	1.1	0.78	0.94
B6 insight	1.7	0.65	1.5	0.70	0.18
B7 quality of social overtures	1.1	0.57	1.0	0.43	0.43
B8 quality of social response	1.2	0.50	1.1	0.44	0.60
B9 amount of reciprocal social communication	0.79	0.71	0.65	0.68	0.41
B10 overall quality of rapport	0.95	0.52	1.0	0.59	0.54
C1 imagination/creativity	0.79	0.63	0.72	0.65	0.67
D1 unusual sensory interest in play material/person	0.74	0.81	0.68	0.74	0.78
D2 hand and finger and other complex mannerisms	0.68	0.82	0.43	0.71	0.16
D3 self-injurious behavior	0.053	0.23	0.063	0.34	0.90
D4 excs inter/ref to unus/highly spec topics/objts/rep behav	0.47	0.61	0.84	0.78	0.056
D5 compulsions or rituals	0.58	0.61	0.54	0.67	0.82
E1 overactivity/agitation	0.16	0.50	0.26	0.50	0.41
E2 tantrums, aggression, negative or disruptive behavior	0.053	0.23	0.12	0.38	0.47
E3 anxiety	0.16	0.50	0.17	0.48	0.91

**Table 10:** ADOS module 4 sub-scores dependent on soy-based formula.

Males	Soy (N=6)		Non Soy (N=29)		
	Avg	SD	Avg	SD	*P*
A4 stereotyped/idiosyncratic use of words/phrases	0.67	0.52	1.2	0.71	0.11
A8 conversation	0.5	0.55	0.97	0.68	0.13
B3 language production and linked nonverbal communication	0	0	0.069	0.26	0.52
B7 insight	1.2	0.75	1.1	0.72	0.85
B10 quality of social response	0.83	0.41	1.1	0.37	0.17
B11 amount of reciprocal social communication	0.5	0.84	0.79	0.68	0.36
**Females**	**Soy (N=0)**		**Non Soy (N=2)**		
	**Avg**	**SD**	**Avg**	**SD**	***P***
A4 stereotyped/idiosyncratic use of words/phrases	---	---	0.50	0.71	---
A8 conversation	---	---	0.5	0.71	---
B3 language production and linked nonverbal communication	---	---	0	0	---
B7 insight	---	---	1.5	0.71	---
B10 quality of social response	---	---	1.0	0	---
B11 amount of reciprocal social communication	---	---	0.5	0.71	

**Table 11:** Summary of line item behaviors altered in response to soy formula.

**ABC**	
Cries over minor hurts	males worse
Prefers to be alone	females worse
Uncooperative	females better
**ADI-R**	
Loss of communicative intent	males worse
	females worse
Comprehension of simple language-most abnormal	males worse
Head shaking-most abnormal	males better
Direct gaze-most abnormal	females better
Showing and direct attention-current	males worse
Offering comfort-current	females worse
Quality of social overtures-current	males worse
Inappropriate facial expression-current	males worse
Unusual preoccupations-current	males worse
Compulsions/rituals-current	males worse
Unusual sensory interests-current	males worse
Undue general sensitivity to noise-current	males worse
Abnormal, idiosyn, negative response to spec sensory stim-current	males worse
Difficulties with minor changes routines/personal env-current males worse	males worse
Difficulties with minor changes routines/personal env-ever males worse	males worse
Other complex mannerisms or stereotyped body movements-current	males worse
Self-injury-current	males worse
**ADOS**	
**Module 1**	
Frequency vocalization directed to others	males worse
Pointing	males worse
Requesting	males worse
Response to joint attention	males worse
Giving	females worse
Spontaneous joint attention	females better
**Module 2**	
Quality of social overtures	males worse
Imagination/creativity	males worse
Amount of social overtures/maintenance of attention	females better
Pointing	females better
Descriptive, conventional, instrumental or informal gestures	females better
Spontaneous initiation of joint attention	females better
Overall quality of rapport	females better
Anxiety	females worse
**Module 3**	
Offers information	males better females worse
Asks for information	males better
Reporting of events	males better
Insight	males better
Descriptive, conventional, instrumental or inform gestures	females worse

## Abbreviations

ABC: Aberrant Behavior Checklist
ADI-R: Autism Diagnostic Interview-Revised
ADOS: Autism Diagnostic Observation Schedule
ASD: Autism Spectrum Disorders
ADHD-IV: Attention-Deficit Hyperactivity Disorder-IV
CERHR: Center for Evaluation of Risks to Human Reproduction
CSI: Communication and Social Interaction
FXS: fragile X syndrome
GARS: Gilliam Autism Rating Scale
NTP: National Toxicology Program
RRB: Restricted and Repetitive Behavior
RRSB: Total Restricted, Repetitive and Stereotyped Behavior
SFARI: Simons Foundation Autism Research Initiative
SSC: Simons Simplex Collection
TSH: Thyroid-Stimulating Hormone
